# Histamine impairs midbrain dopaminergic development *in vivo* by activating histamine type 1 receptors

**DOI:** 10.1186/s13041-014-0058-x

**Published:** 2014-08-12

**Authors:** Itzel Escobedo-Avila, Fernanda Vargas-Romero, Anayansi Molina-Hernández, Rodrigo López-González, Daniel Cortés, Juan A De Carlos, Iván Velasco

**Affiliations:** 1 Instituto de Fisiología Celular-Neurociencias, Universidad Nacional Autónoma de México, México D.F.-04510, Mexico; 2 Current address: Instituto Nacional de Perinatología “Isidro Espinosa de los Reyes”, México D.F.-11000, Mexico; 3 Instituto Cajal, CSIC, Madrid 28002, Spain

**Keywords:** Neural stem cells, Midbrain development, Dopaminergic neurons, Ultrasound injections, Parkinson’s disease

## Abstract

**Background:**

Histamine (HA) regulates the sleep-wake cycle, synaptic plasticity and memory in adult mammals. Dopaminergic specification in the embryonic ventral midbrain (VM) coincides with increased HA brain levels. To study the effect of HA receptor stimulation on dopamine neuron generation, we administered HA to dopamine progenitors, both *in vitro* and *in vivo*.

**Results:**

Cultured embryonic day 12 (E12) VM neural stem/progenitor cells expressed transcripts for HA receptors H_1_R, H_2_R and H_3_R. These undifferentiated progenitors increased intracellular calcium upon HA addition. In HA-treated cultures, dopamine neurons significantly decreased after activation of H_1_R. We performed intrauterine injections in the developing VM to investigate HA effects *in vivo*. HA administration to E12 rat embryos notably reduced VM Tyrosine Hydroxylase (TH) staining 2 days later, without affecting GABA neurons in the midbrain, or serotonin neurons in the mid-hindbrain boundary. qRT-PCR and Western blot analyses confirmed that several markers important for the generation and maintenance of dopaminergic lineage such as TH, Lmx1a and Lmx1b were significantly diminished. To identify the cell type susceptible to HA action, we injected embryos of different developmental stages, and found that neural progenitors (E10 and E12) were responsive, whereas differentiated dopaminergic neurons (E14 and E16) were not susceptible to HA actions. Proliferation was significantly diminished, whereas neuronal death was not increased in the VM after HA administration. We injected H_1_R or H_2_R antagonists to identify the receptor responsible for the detrimental effect of HA on dopaminergic lineage and found that activation of H_1_R was required.

**Conclusion:**

These results reveal a novel action of HA affecting dopaminergic lineage during VM development.

## Background

Histamine (HA) is a neurotransmitter that participates in the sleep cycle, motor activity, synaptic plasticity and memory [1]–[5]. The adult mammalian brain contains somas of HA-producing neurons in the hypothalamic tuberomamillary nucleus, and its projections reach most of the CNS [3],[4],[6]. HA exerts its actions through the activation of four G-protein-coupled receptors: H_1_R-H_4_R. H_1_R, H_2_R and H_3_R are widely distributed throughout the CNS [6]–[8], but the presence of H_4_R in the brain remains unclear [9]–[11].

During embryonic development, HA is present at higher concentrations than those observed in the adult brain [12]–[14]. HA increases its content at embryonic days (E) 14–16, and then decreases perinatally [13],[15]–[17]. Histidine decarboxylase (HDC), which synthetizes HA, and the mRNAs for H_1_R, H_2_R and H_3_R [18]–[21] are also expressed at these developmental stages [20],[22],[23]. The transitory expression of HDC in the midbrain, together with elevated HA levels, and the expression of HRs, coincides with periods of dopaminergic specification [24], suggesting that HA might influence dopamine (DA) neuron generation in the ventral midbrain (VM). In rats, differentiated dopaminergic neurons are first identified at E12 by expression of Tyrosine Hydroxylase (TH), the rate-limiting enzyme for DA synthesis. The peak of DA neurogenesis is at E13. By E16, all DA cells are post-mitotic and their axons are *en route* to reach forebrain structures such as the striatum and the cerebral cortex [25],[26].

On cortical neural stem/progenitor cell (NSPC) cultures, the effect of activating H_1_ and H_2_ receptors has been reported [27]. HA increases cell proliferation by H_2_R activation and has a neuronal-differentiating action mediated by H_1_R stimulation, probably by rising *prospero 1* and *neurogenin 1* expression [28] and favoring differentiation to FOXP2-positive neurons [29]. Furthermore, HA stimulates neuronal differentiation of adult subventricular zone NSPC [30]. The HA concentrations required to observe these effects are 100 μM to 1 mM.

Elucidating the developmental pathways that control neuronal specification in the VM is of great relevance to increase our knowledge in differentiation of dopaminergic cells. In this work, we aimed to study the effect of HA on dopaminergic development. We found that HA is detrimental to dopaminergic differentiation of NSPC *in vitro*, and demonstrate that HA administration *in vivo* at early developmental stages decreases dopaminergic induction in the VM through H_1_R stimulation. This study establishes the inhibitory relationship of HA to DA neuron generation during development, and provides a novel mechanism for the future treatment of Parkinson’s disease.

## Results

### Midbrain NSPC cultures are multipotent and express histaminergic receptors

**Figure 1:**
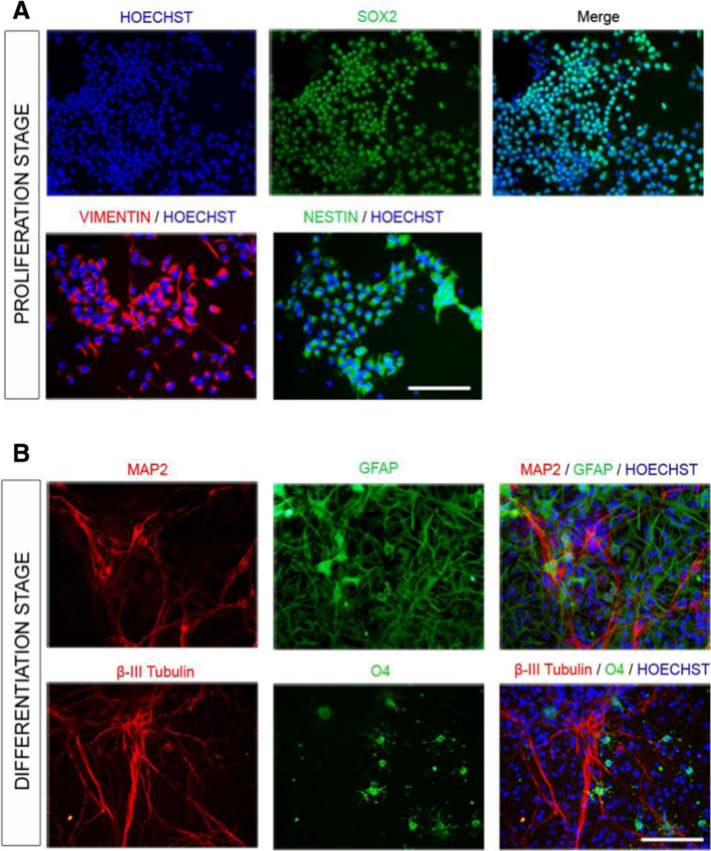
**Ventral midbrain NSPC isolated from E12 rat embryos are multipotent. (A)** VM NSPC cultured in the presence of the mitogenic factor FGF-2 express the markers of undifferentiated cells: Sox2, Vimentin and Nestin in proliferation stage. **(B)** After 6 days without FGF-2 (differentiation stage), VM NSPC differentiate into the three lineages of Central Nervous System: neurons (β-III Tubulin + and MAP2+), astrocytes (GFAP+) and oligodendrocytes (O4+), confirming the multipotency of these cultures. Nuclei were stained with Hoechst. Scale bars = 100 μm.

NSPC have the capacity to self-renew, and the potential to differentiate into neurons, astrocytes and oligodendrocytes. To characterize the expression of NSPC markers, as well as their capacity to differentiate to neuronal and glial populations, we cultured VM NSPC isolated from E12 rat embryos. We maintained these cells in proliferation during 4 days in the presence of the mitogen Fibroblast growth factor (FGF)-2, and then induced differentiation for 6 days after removal of FGF-2. We found that a very high proportion of these cells express Sox2, Vimentin and Nestin, which are markers widely used to identify undifferentiated NSPC (Figure 1A). After removing FGF-2 from cultures, cells readily differentiate into neurons (MAP2- and β-III Tubulin-positive), astrocytes (Glial Fibrillary Acidic Protein, GFAP-positive) and oligodendrocytes (O4-positive) (Figure 1B), confirming that our cultures are indeed NSPC.

**Figure 2:**
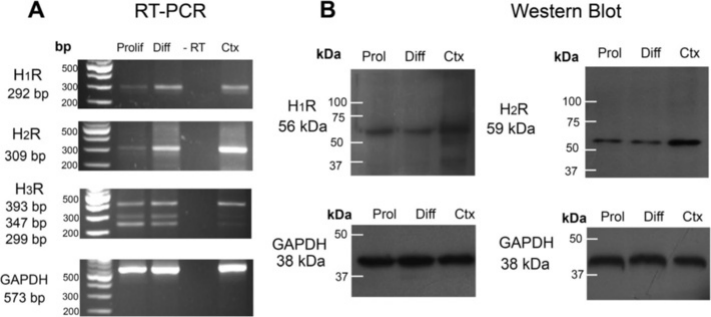
**Histaminergic receptors are expressed in rat NSPC ventral midbrain cultures. (A)** RT-PCR experiments to detect H_1_, H_2_ and H_3_ receptor mRNAs in proliferating (Prolif) and differentiated (Diff) cultured cells from E12 VM. One μg of total RNA was used for reverse transcription. The expression of gene transcripts for H_1_, H_2_ and H_3_ receptors were analyzed by electrophoresis on a 2% agarose gel. Rat adult cerebral cortex (Ctx) was used as positive control and RNA without retrotranscriptase (−RT) as negative control. **(B)** Western blot experiments performed to detect H_1_ and H_2_ receptors in E12 midbrain proliferating and differentiated cells. Rat adult cerebral cortex (Ctx) was used as positive control. Thirty μg of protein were resolved on 8% SDS-PAGE, transferred to nitrocellulose membranes, and probed with anti-HA receptors antibodies. Immunoreactive bands were detected by enhanced chemiluminescence. In RT-PCR and Western blot experiments, GAPDH was used as control.

Previous work has demonstrated that mRNAs for histaminergic receptors are present in rodent embryos from E14 onwards [18],[20],[21], but no information has been reported regarding the expression of these receptors at earlier stages of embryogenesis neither in the VM tissue, nor in midbrain NSPC *in vitro*. Therefore, using primers verified to amplify HA receptors [27], we examined whether these receptors were present in cultured VM NSPC, before and after differentiation. We found by RT-PCR that proliferative and differentiated VM NSPC cultures, isolated from E12 rat embryos, express transcripts of H_1_R, H_2_R, and three isoforms reported for the H_3_R, which are generated by alternative splicing [19],[21],[31],[32] (Figure 2A). The VM tissue without culture also had mRNAs for H_1_, H_2_ and H_3_ receptors (data not shown). We also performed Western blot analyses and found that the corresponding proteins for H_1_R and H_2_R were present in cultured NSPC (Figure 2B) and in VM tissue (data not shown). Even when the level of mRNA for H_1_ and H_2_ receptors was low, a single band was clearly detected, showing that H_1_R and H_2_R proteins are present in E12 cultured VM NSPC.

### HA increases intracellular calcium in VM NSPC

**Figure 3:**
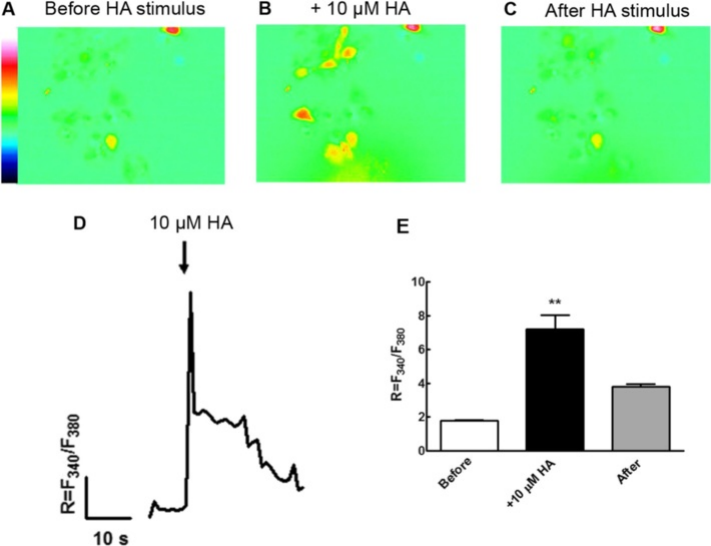
**HA stimulation increases intracellular Ca**^**2+**^**in undifferentiated VM NSPC.** Fluorescence images of responsive VM NSPC showing variations of [Ca^2+^]_i_**(A)** before, **(B)** during and **(C)** after stimulation with 10 μM HA. **(D)** Average ratio (R) values of fluorescence resulting from alternating excitation of Fura-2 between 340 nm and 380 nm (R = F_340_/F_380_) are shown. **(E)** A 3-fold increase was found in proliferating VM NSPC exposed to 10 μM HA. **p < 0.01.

Calcium (Ca^2+^) is an important regulator of various neuronal functions. During the formation of CNS, Ca^2+^ signaling is involved in the regulation of processes such as neural induction and differentiation of neural progenitors into neurons and glia, in a defined spatiotemporal pattern [33],[34]. Since the activation of H_1_R leads to the accumulation of Ca^2+^ as a second messenger, we tested if undifferentiated VM NSPC were responsive to HA stimulation by elevating intracellular Ca^2+^ concentration ([Ca^2+^]_i_). To address this hypothesis, we loaded cultured Nestin + VM NSPC with the ratiometric calcium probe Fura-2 AM, and measured the fluorescence changes resulting from alternating excitation of Fura-2 between 340 nm and 380 nm (R = F_340_/F_380_). We found a significant 3-fold increase in [Ca^2+^]_i_ after the addition of 10 μM HA to VM NSPC, compared to the basal levels observed in control conditions (Figure 3), indicating that undifferentiated VM NSPC are responsive to HA. These results are consistent with our previous work on cerebrocortical NSPC [29], where 100 μM HA also caused calcium increases in VM cells through H_1_R activation.

### HA increases apoptotic cell death of cultured undifferentiated midbrain NSPC without affecting cell proliferation

**Figure 4:**
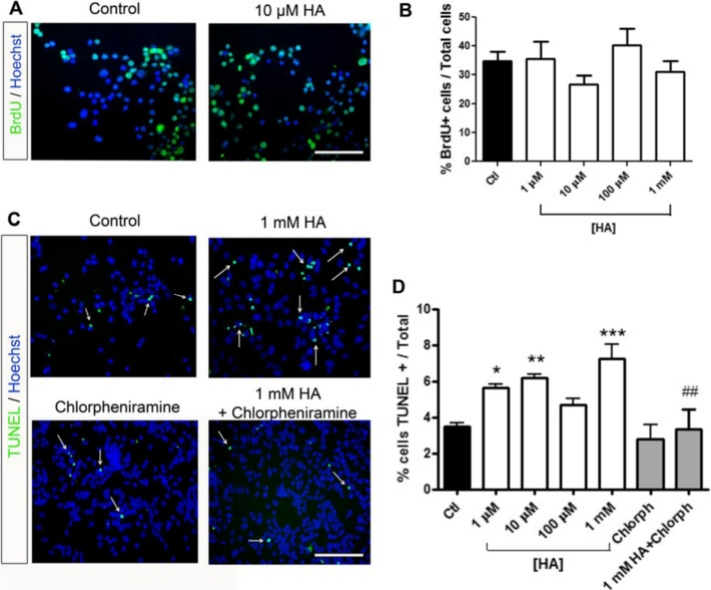
**HA does not alter BrdU incorporation but increases apoptotic cell death of proliferating VM NSPC by activating H**_**1**_**R.** The effect of HA on proliferation and apoptotic cell death was evaluated by BrdU incorporation and TUNEL assays, respectively. **(A)** Micrographs showing immunostaining to detect BrdU-positive cells. Hoechst was used for visualization of nuclei. **(B)** Percentage of BrdU-positive cells. No statistically significant differences were found. **(C)** Micrographs showing TUNEL-positive cells (white arrows) and Hoechst-stained nuclei. One mM HA caused a significant increase in apoptotic cells, which was prevented by chlorpheniramine. **(D)** Percentage of the total cell number of TUNEL-positive cells. One millimolar HA-treatment increased the proportion of apoptotic cells compared to control condition. *p < 0.05; **p < 0.01; ***p < 0.001. The H_1_R antagonist chlorpheniramine significantly reverted this effect. ^##^p < 0.01 compared to 1 mM HA. Scale bars = 100 μm.

In order to examine the effects of HA on VM NSPC proliferation and apoptotic cell death, we treated cultures with increasing concentrations of HA (from 1 μM to 1 mM) in the presence of FGF-2 for 4 days. We used BrdU incorporation assays to analyze proliferation and the TUNEL assay to analyze apoptotic cell death. We calculated the percentage of BrdU- and TUNEL-positive cells relative to the total number of cells in control and HA-treated cultures and found that HA did not affect BrdU incorporation (Figure 4A and B). In contrast, 1 mM HA significantly increased the proportion of TUNEL-positive cells and this effect was prevented by co-incubation with the H_1_R antagonist chlorpheniramine (Figure 4C and D).

### HA decreases the number of dopaminergic neurons in differentiated VM NSPC

**Figure 5:**
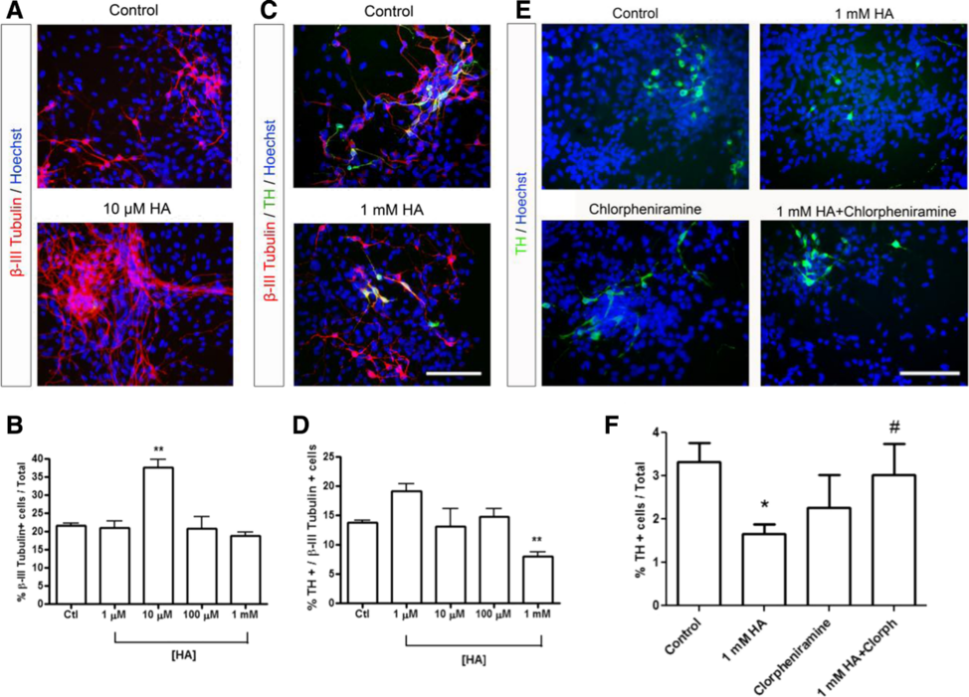
**HA promotes neuronal differentiation but decreases dopaminergic neurons in cultured VM NSPC.** After proliferation, cells were kept on differentiating conditions for 6 days (N2 medium without FGF-2) and treated daily with different concentrations of HA (from 1 μM to 1 mM HA). VM NSPCs were analyzed after differentiation. **(A)** Micrographs showing the neuronal marker β-III Tubulin in control and 10 μM HA-treated cultures. Nuclei were stained with Hoechst. **(B)** Quantification of β-III Tubulin labeled cells relative to the total number of cells, showing a significant increase in the proportion of β-III Tubulin + cells caused by treatment with 10 μM HA. **(C)** Micrographs of double immunocytochemistry to detect the neuronal marker β-tubulin III and the dopaminergic marker Tyrosine Hydroxylase (TH) in control and 1 mM HA-treated cultures. Nuclei were stained with Hoechst. Scale bar = 100 μm. **(D)** Graph showing the percentage of TH-positive neurons in control and after HA treatments, relative to the total number of β-Tubulin III-positive cells. TH + neurons were significantly decreased after treatment with 1 mM HA. **p < 0.01. **(E)** Micrographs showing the dopaminergic marker TH in control cells, and the decrease caused by 1 mM HA. The H1R antagonist chlorpheniramine was tested either with or without HA. Nuclei were stained with Hoechst. **(F)** HA-induced decrease of TH-positive numbers was antagonized by chlorpheniramine. Note that the percentage of TH-positive cells is lower than in Figure 4D because it is normalized by the total number of cells. *p < 0.05 relative to control; ^##^p < 0.01 compared to 1 mM HA. Scale bars = 100 μm.

To analyze whether HA has an effect on the differentiation of VM NSPC, cultures were treated for 6 days in the absence of FGF-2. Then, we evaluated by immunocytochemistry the expression of the neuronal marker β-III Tubulin. We found that 10 μM HA significantly increased the percentage of β-III Tubulin -positive cells from 21% to 37%, compared to control (Figure 5A and B). Additionally, we analyzed whether HA could have a role on dopaminergic differentiation by detecting the expression of the rate limiting enzyme for dopamine synthesis, TH, normalized by the number of neurons positive to β-III Tubulin. Figure panels 5C and D show that 1 mM HA significantly reduced the proportion of dopaminergic neurons when compared to controls. To establish if the decrease on TH-positive neurons was mediated by H_1_R activation, we treated cultures with chlorpheniramine, alone or in combination with 1 mM HA. We observed a significant recovery in the number of TH + cells when HA plus chlorpheniramine were added, compared to HA alone (Figure 5E and F).

### In vivo experiments

#### Intrauterine administration of HA does not modify the morphology of the VM

**Figure 6:**
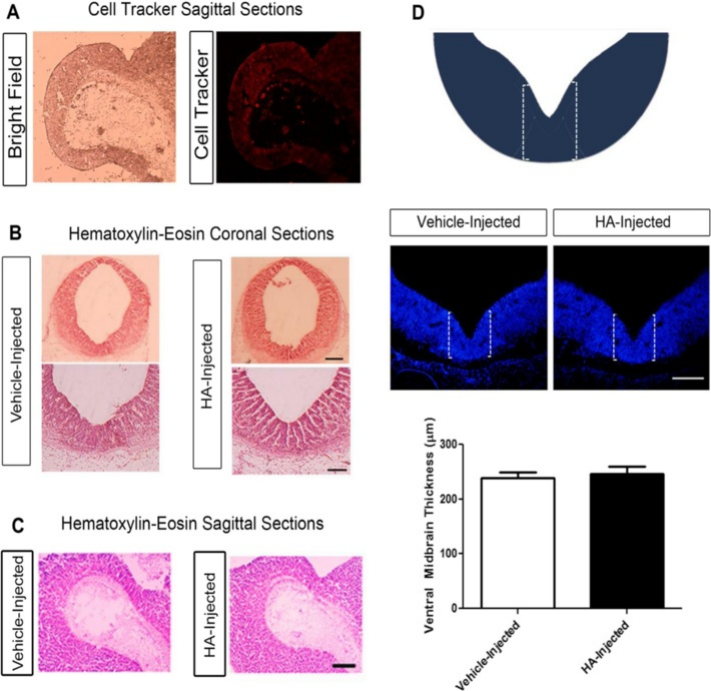
**Intrauterine injections reached the VM and do not alter the midbrain morphology. (A)** Bright field and fluorescent images of an E14 brain injected in E12 with cell tracker and HA. **(B)** Hematoxylin and eosin staining of E14 ventral midbrain (VM) coronal sections of vehicle-injected or HA-injected embryos. **(C)** Comparison of E14 VM sagittal sections stained with hematoxylin and eosin from vehicle- and HA-injected embryos, where no histological differences were found. Scale bars: 500 μm and 150 μm. **(D)** To more quantitatively assess the VM, measurements of the thickness corresponding to the ventral part of the midbrain, where dopaminergic neurons are present, were performed; the brackets mark the regions used to measure thickness in both sides of the brain. The graph represents the average of thickness in both sides. No difference between vehicle- and HA-injected embryos was found. Scale bar: 150 μm.

In order to test if the effects of HA observed *in vitro* were also present in the developing brain *in vivo*, we performed *in utero* microinjections of HA dihydrochloride, which was used without neutralization. In these experiments, vehicle, HA or its receptor’s antagonists were injected directly into the ventricular lumen of E12 rat embryos. This stage was selected because it precedes the peak of neurogenesis in the midbrain. We initially injected 25 μg of HA and did not observe any change in neuronal differentiation relative to vehicle-injected embryos. We then administered 50 μg of HA, and analyzed the brains at E14. To evaluate if HA injection reached the VM region, we co-injected HA with a fluorescent tracer (Cell tracker), and found that the injected volume was enough to cover consistently the whole midbrain neuroepithelium (Figure 6A). There were no morphological differences between vehicle- and HA-injected embryos, assessed by hematoxylin-eosin staining in both coronal (Figure 6B) and sagittal (Figure 6C) sections of VM. To assess a general alteration of the VM, where TH-positive neurons are generated, the thickness of this region was measured bilaterally. No significant differences on the average VM thickness were found between vehicle- and HA-injected embryos (Figure 6D).

#### HA injection decreases dopaminergic induction in VM

**Figure 7:**
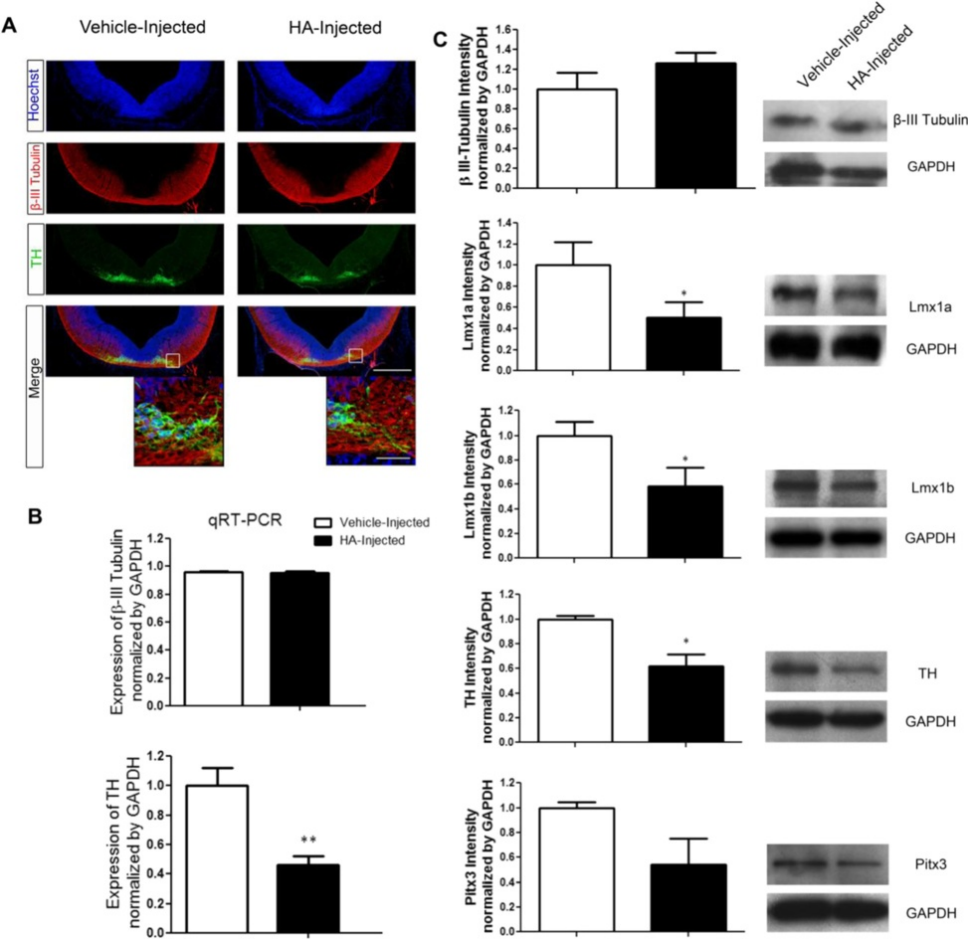
**HA injection decreases dopaminergic neurons without affectingβ-III Tubulin. (A)** Coronal sections of the ventral midbrain from E14 vehicle- and HA-injected rat embryos stained to detect neurons (β-III Tubulin+) and dopaminergic phenotype (TH+); a representative section of a vehicle-injected embryo showing many TH-positive neurons while in a similar midbrain section from a representative HA-injected embryo less TH-positive cells are present. Nuclei were detected with Hoechst. Inner white squares in the merge images represent the higher magnifications shown at the bottom. Scale bars: 150 μm and 50 μm for the low-power and high-power pictures, respectively. **(B)** β-III Tubulin and TH expression by qRT-PCR from E14 vehicle- and HA-injected embryos, with β-III Tubulin presenting no change, and a significant decrease in TH caused by HA. **p < 0.01. **(C)** Densitometric analyses of the effect of HA administration on protein levels of β-III Tubulin and diverse factors involved in dopaminergic specification and phenotype. HA injection decreased the protein level of TH, Lmx1a, Lmx1b and Pitx3 in midbrain tissue from E14 HA-injected embryos without affecting the generation of neurons, compared to the vehicle-injected condition. Values were normalized to GAPDH signal. *p < 0.05.

Since we found that HA induced a decrease in TH + neurons after differentiation of cultured NSPC, we inquired whether this effect is also present *in vivo*. For this purpose, E12 rat embryos were injected in the cerebroventricular lumen either with vehicle or HA. Embryos were allowed to develop and then euthanized at E14. HA injection produced no decrease in TH staining without affecting β-III Tubulin-positive neurons (Figure 7A). These results suggest that HA is not interfering with neuronal differentiation since the normal pattern of β-III Tubulin-positive neurons was unaffected. In order to more quantitatively assess neuronal differentiation and the decline in dopaminergic neurons, β-III Tubulin and TH mRNA expression was evaluated by quantitative RT-PCR in vehicle- and HA-injected embryos. HA caused a significant decline in TH transcripts without affecting β-III Tubulin expression (Figure 7B). In addition, we analyzed by Western blot β-III Tubulin, TH and other markers specific for VM dopaminergic phenotype. HA induced a marked reduction in TH, Lmx1a, Lmx1b and Pitx3 protein levels relative to vehicle (Figure 7C). These experiments strongly support the notion that HA interferes with dopaminergic neuronal generation without modifying the proportion of neurons in the VM.

#### Injection of HA does not alter GABA or serotonin neurons

**Figure 8:**
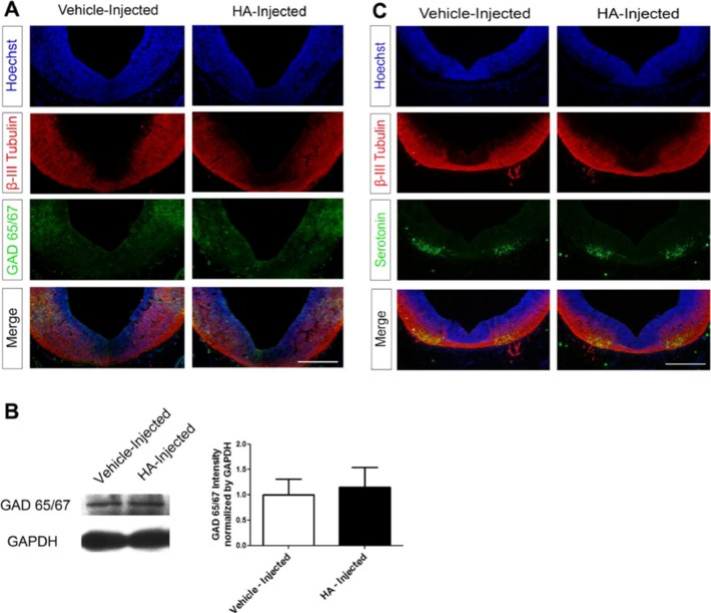
**HA does not alter GABAergic or serotoninergic neurons. (A)** Coronal sections of the ventral midbrain from E14 vehicle- and HA-injected rat embryos stained to identify neurons (β-III Tubulin+) and GABA-synthesizing cells (anti-GAD65/67+ antibody); vehicle-injected embryos had the same proportion of GAD65/67+ cells when compared to HA-injected embryos. Nuclei were stained with Hoechst. Scale bar: 150 μm. **(B)** HA did not modify the level of GAD65/67 protein in E14 midbrain tissue compared to vehicle-injected embryos by Western blot. The graph represents the densitometric analysis of GAD65/67 protein content where no significant effects were found; values were normalized to GAPDH. **(C)** Coronal sections of the ventral midbrain from vehicle- and HA-injected E14 embryos stained with β-III Tubulin and serotonin antibodies, where no differences in serotoninergic neurons are observed. Scale bar: 150 μm.

We also analyzed other neuronal phenotypes present in the developing brain in embryos injected at E12. GABAergic neurogenesis in the ventral and the dorsal regions of the midbrain begins simultaneously with dopaminergic neurogenesis [35]. GABAergic neurons express GAD65 and/or GAD67; GAD enzymes decarboxylate glutamate to produce GABA. Labeling of GAD65/67 with an antibody that recognizes both isoforms did not change after HA administration, when evaluated by immunohistochemistry (Figure 8A) and immunoblot (Figure 8B). The GAD staining pattern is restricted to the dorso-lateral regions of the midbrain compared to the ventral-most localization of dopaminergic neurons. The presence of serotoninergic neurons was identified by immunostaining against serotonin. The distribution of these neurons with or without HA was found to be in their normal arrangement in the most posterior part of the midbrain, close to the hindbrain, the cerebral regions where most serotoninergic neurons are generated (Figure 8C).

#### HA interferes with dopaminergic development only when administered at early stages

**Figure 9:**
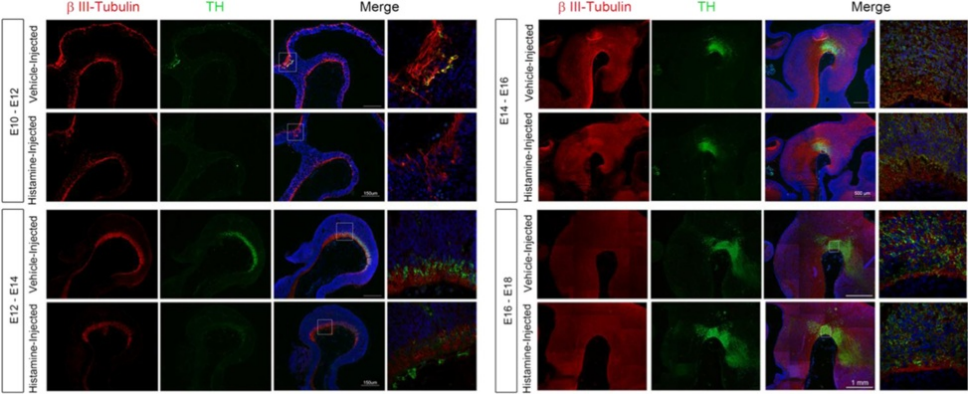
**HA acts on early dopamine neural precursors but differentiated dopamine neurons are resistant.** Sagittal sections of the VM from vehicle- and HA-treated rat embryos that were injected at different developmental stages, and analyzed two days later. Sections were stained to detect neurons (β-III Tubulin+) and dopaminergic phenotype (TH+); nuclei were detected with Hoechst. The decreasing effect of HA injection on dopaminergic phenotype is evident only when injected at early developmental stages (E10-E12 or E12-E14) but not at later developmental stages (E14-E16 or E16-E18). For the vehicle-injected E10-E12 embryos, only a few TH + neurons were found in the isthmic region and these neurons were absent in HA-injected organisms. Scale bars are indicated in the figure.

In order to establish whether embryos of different developmental stages were equally susceptible to HA actions as we observed at E14, we performed injections at E10, E12, E14 or E16 and analyzed TH expression in VM two days later in every case. We found that when HA was injected at E10 and E12, a marked decrease in TH staining was observed. In contrast, when HA was administered at E14 or E16, the TH expression pattern was indistinguishable from vehicle-injected embryos (Figure 9), strongly suggesting that HA is acting on neural precursors, and not on differentiated neurons.

#### HA decreases cell proliferation without altering cell death

**Figure 10:**
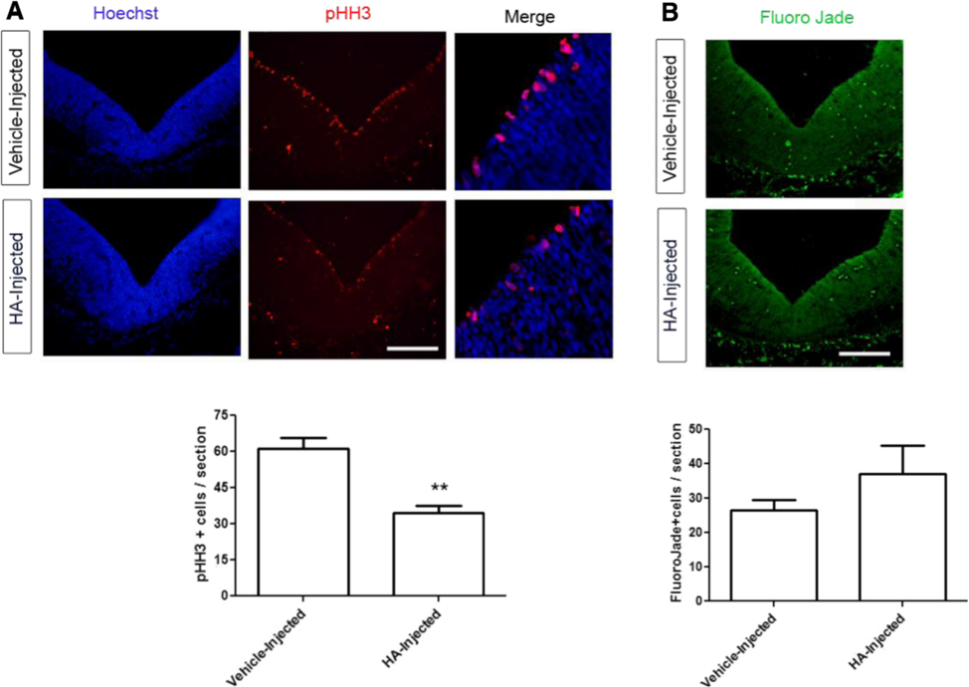
**HA diminishes proliferation without altering cell death in VM. (A)** Proliferation was assessed by staining with the mitotic marker pHH3 in coronal sections of the ventral midbrain from E14 vehicle- and HA-injected embryos. HA-injected embryos had significantly less pHH3+ cells when compared to vehicle-injected embryos. Nuclei were stained with Hoechst. *p < 0.05. **(B)** Coronal VM sections showing Fluoro Jade staining where no significant differences were observed in degenerating cells. Scale bar: 150 μm.

After we found that HA induced a decrease in TH + neurons *in vivo* at early developmental stages*,* we asked whether this effect was produced by means of decreased proliferation or increased cell death. We injected embryos at E12 and coronal sections of vehicle- and HA-injected E14 embryos were stained with anti-phosphorylated histone H3 (pHH3), a marker for cells in M-phase. The pHH3 staining pattern of vehicle-injected embryos is consistent with previous reports [36]. However, the injection of HA caused significantly less pHH3-positive cells in the VM compared with vehicle-injected embryos (Figure 10A). We assessed apoptotic cell death *in vivo* and found no significant differences caused by HA administration (data not shown). We then decided to use Fluoro Jade staining to detect neuronal death, and found that HA injection did not modify the proportion of degenerating cells (Figure 10B). Thus, our results show that HA is affecting proliferation *in vivo* without modifying cell death in the VM.

#### The effect of HA on dopaminergic differentiation is mediated by H_1_R

**Figure 11:**
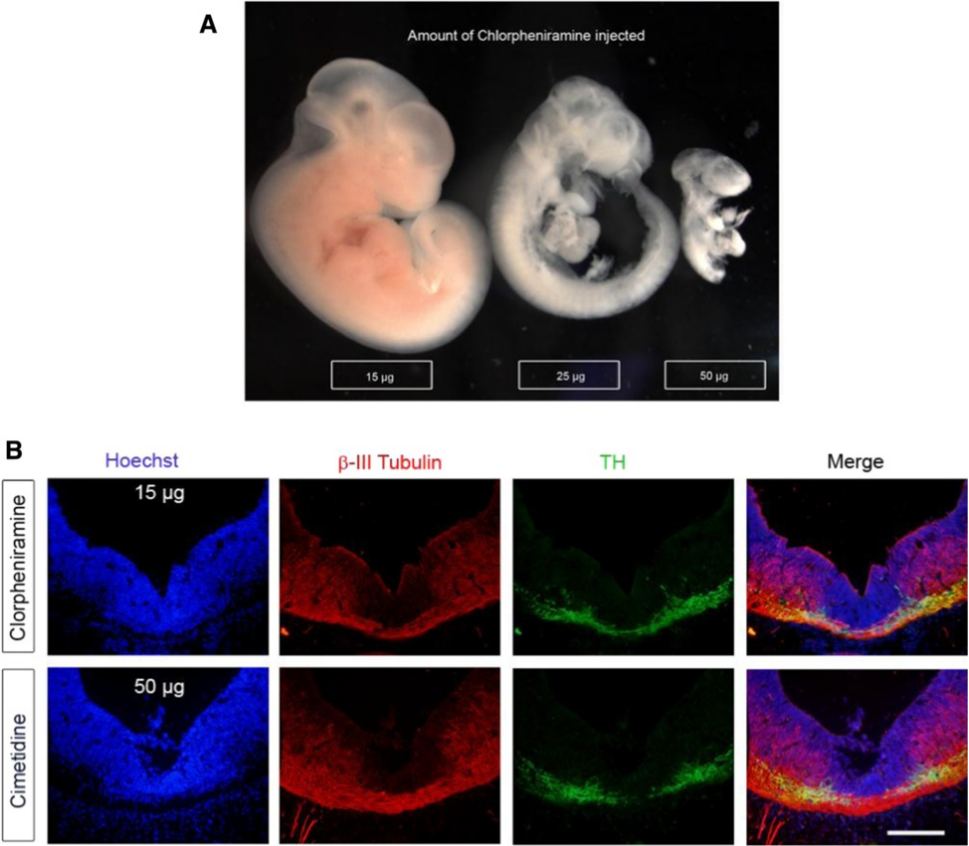
**High doses of chlorpheniramine affect normal embryonic development but neither chlorpheniramine nor cimetidine affects dopaminergic neurons. (A)** Micrographs from E14 rat embryos injected at E12 with different doses of the H_1_R antagonist, chlorpheniramine. Fifty and twenty-five micrograms interfered with normal development, while 15 μg allows normal development of the embryo. **(B)** TH and β-III Tubulin staining in coronal sections of the VM from E14 rat embryos injected with 15 μg of the H_1_R antagonist, chlorpheniramine or 50 μg of the H_2_R antagonist, cimetidine. The staining patterns were not modified by these antagonists. Scale bar: 150 μm.

**Figure 12:**
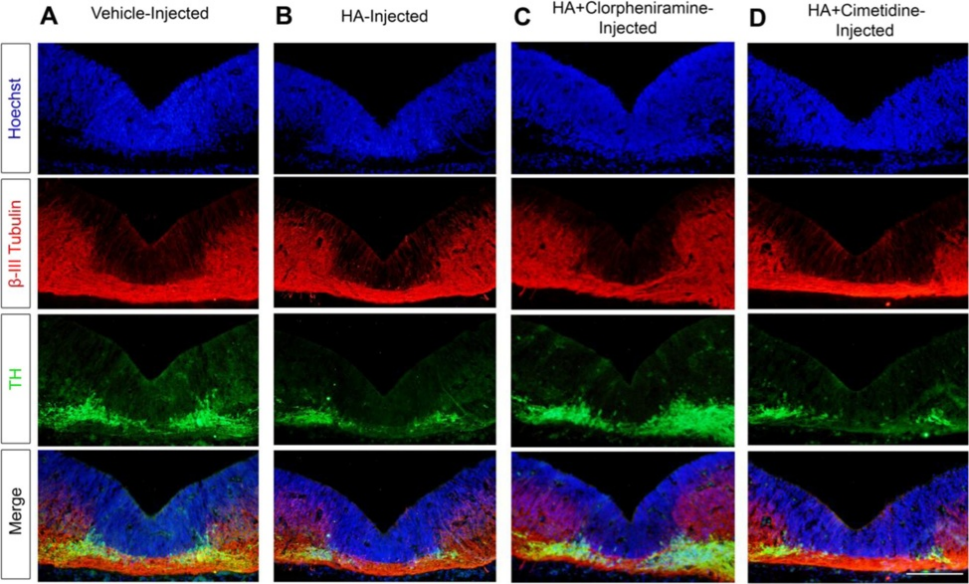
**The H**_**1**_**R antagonist chlorpheniramine, but not the H**_**2**_**R antagonist cimetidine, abolished the deleterious effect of HA administration on dopaminergic differentiation. (A-D)** TH and β-III Tubulin staining in coronal sections of the VM from E14 rat embryos with the indicated treatments. **(A)** Normal pattern of dopaminergic neuron staining in a vehicle-injected embryo. **(B)** Decrease in TH immunoreactivity due to HA administration. **(C)** Protective effect of the H_1_R antagonist chlorpheniramine on HA-induced decrease of TH immunoreactivity. **(D)** Administration of HA and the H_2_R antagonist cimetidine did not modify dopaminergic neurons compared to HA-injected embryos. Scale bar: 150 μm.

In order to identify which receptor was responsible for the observed HA effects, injections of H_1_R or H_2_R antagonists, alone or in combination with HA, were performed. We analyzed the TH staining pattern in VM coronal sections of E14 embryos injected 2 days before. The H_1_R antagonist chlorpheniramine, injected at high doses (25 μg or 50 μg), markedly affected the normal development of the embryos, causing non-specific effects. When we injected 15 μg of chlorpheniramine, we observed a normal morphology of the treated embryos (Figure 11A). This latter dose of chlorpheniramine did not affect the expression of TH (Figure 11B); however, when co-injected with HA (Figure 12C), chlorpheniramine blocked HA-induced decrease in TH (Figure 12B), resulting in a TH staining similar to vehicle-injected embryos (Figure 12A). Cimetidine, a specific H_2_R antagonist, at the tested dose (50 μg), did not modify the normal embryo development nor TH staining, when injected without HA (Figure 11B). Co-injection of cimetidine with HA did not antagonize the effect triggered by HA (Figure 12D). These results demonstrate that the decrease of TH + cells is mediated through the activation of H_1_R.

## Discussion

In this study we report that: i) midbrain NSPC express HA receptors; ii) HA stimulus increases intracellular calcium in proliferative VM NSPC; iii) one millimolar HA promotes apoptotic death of cultured VM NSPC by activating H_1_R; iv) Ten μM HA increases neuronal differentiation, but 1 mM HA decreases the number of differentiated dopaminergic neurons, in a H_1_R-dependent fashion, on VM NSPC cultures; v) HA injection at E12 decreases dopaminergic induction in VM without altering GABA or serotonin neurons *in vivo*; vi) HA interferes with dopaminergic development only when administered at early developmental stages; vii) HA impaired cell proliferation in the VM *in vivo* and viii) the effect of HA on dopaminergic differentiation is mediated by activation of H_1_R.

We found that H_1_ and H_2_ receptors are present at both mRNA and protein levels in proliferating and differentiated cultured VM NSPC. We showed that upon HA stimulation, undifferentiated cultures of VM NSPC significantly increased the cytoplasmic Ca^2+^ concentration. This data strongly suggest the presence of functional H_1_R coupled to the canonical calcium response in VM NSPC, consistent with the calcium responses evoked by 100 μM HA in cortical NSPC, which is blocked by chlorpheniramine [29], supporting the notion that H_1_R activation in undifferentiated NSPC occurs in both the forebrain and midbrain. It is well established that intracellular Ca^2+^ regulates neurogenesis as well as neurite/nerve growth and axonal pathfinding [34],[37],[38]. In cortical NSPC, H_1_R was responsible for the increased neuronal differentiation caused by HA. We explored the effect of HA on proliferation, cell death and differentiation of cultured midbrain NSPC. No significant differences on cell proliferation were found in HA-treated VM NSPC, when compared to control conditions. Regarding cell death, we observed an increasing effect of HA on apoptosis with 1 mM HA, which was clearly prevented by chlorpheniramine. We have previously reported that HA, through H_1_R activation, increases differentiation of E14 cortical NSPC to neurons that are positive for FOXP2, a marker of deep cortical layers [27],[29]. In contrast, in this study we found that in E12 VM NSPC, 10 μM HA increased neurogenesis and 1 mM HA affected DA neuron generation. These results highlight that the effects of HA vary depending on the studied brain region and the concentration of HA used. In VM NSPC, the decrease in TH + cells was mediated by H_1_R.

Previous studies have shown the expression patterns of HA-immunoreactive neurons and HRs at different stages of development [15]. HA-immunoreactive neurons are first detected on E13 in the regions of mesencephalon and metencephalon, this expression pattern remains until E17 and gradually decreases towards birth [15]. The expression patterns of HRs were reported at later stages of development (from E14 onwards) by *in situ* hybridization [18],[20]. We now show by RT-PCR and Western blot the presence of H_1_ and H_2_ receptors in E12 cultured VM NSPC*.*

Ultrasound guided-injection is a valuable technique that allows visualization and manipulation of embryos at specific developmental stages [39]–[41]. We did not find morphological differences between the vehicle- and HA-injected embryos, assessed by hematoxylin-eosin staining and the VM thickness, which allowed us to confidently proceed to analyze the effect of HA on midbrain development. We corroborated, by co-injections with Cell tracker, that HA reached all cells in the VM. When we evaluated neuronal differentiation by immunostaining, qRT-PCR and immunoblot, HA did not modify β-III Tubulin expression, suggesting that HA is not precluding neuronal differentiation. However, consistent with the data in VM NSPC cultures, when the limiting enzyme for DA synthesis, TH, was evaluated by immunohistochemistry, qRT-PCR and Western blot, we found significant decreases after HA application at E12, pointing out to a detrimental effect on dopaminergic neurons *in vivo*. These results suggest that HA might be affecting either DA specification and/or differentiation. To study if DA specification was being affected, we analyzed the expression of the Lim homeodomain genes *Lmx1a* and *Lmx1b*, which are specific markers for midbrain dopaminergic precursors. Lmx1a is an early marker of dividing mesencephalic precursors in the floor plate ventricular cells at early stages of development, instructing their differentiation into the midbrain dopaminergic phenotype, while Lmx1b is essential for the generation of properly differentiated midbrain dopaminergic neurons and its regional specification [42],[43]. HA significantly decreased the expression levels of both markers. Pitx3, a protein important for maintenance of dopaminergic identity, also decreased in HA-injected embryos, relative to vehicle-treated animals. To further support that HA is acting on DA precursors, E10 and E12 embryos were injected either with vehicle or HA. We demonstrated that the impairing effect of HA over the dopaminergic lineage is present in both developmental stages. In sharp contrast, DA neurons were unaffected when HA was applied at E14 or E16, when most cells already differentiated to dopaminergic neurons. These results strongly support that detrimental action of HA is on VM dopaminergic neural precursors rather than early differentiated neurons.

We found that HA did not modify BrdU incorporation in cultured VM NSPC, but significantly decreased the number of ventricular pHH3-positive cells *in vivo*. This different effect of HA on proliferation might be explained by the fact that BrdU was added to cultures for 3 h, and is incorporated by cells in the S phase of cell cycle, whereas pHH3 is present in cells undergoing mitoses. An additional factor to consider is that 100 μM HA caused a significant increase of BrdU incorporation in cultured cortical NSPC in the presence of daily-added 10 ng/ml FGF-2 [27], the same conditions used here for VM NSPC. A potentiation of thymidine incorporation has also been reported for tracheal smooth muscle human cells incubated with 10 ng/ml Epidermal Growth Factor and 10 μM HA, compared to the growth factor-only condition; this increased proliferation was associated to activation of Akt with participation of Gq/11 receptors [44]. It is possible that in cultured VM cells there are subpopulations that differentially respond to HA: some might decrease (i.e. dopaminergic precursors), while others (non-dopaminergic cells) could increase BrdU incorporation, resulting in no overall changes after HA addition. This possibility is supported by the fact that HA caused a significant decrease in the number of differentiated dopaminergic neurons both *in vitro* and *in vivo*.

We also analyzed the differentiation of other neuronal phenotypes present in midbrain-surrounding regions. Both serotoninergic and dopaminergic neurons arise from the ventral-most neuroepithelial progenitors, although at different rostro-caudal levels [45]. GABAergic cells are present in the *substantia nigra pars reticulata* and to some extent within the *substantia nigra pars compacta* and also in the ventral tegmental area [35]. Here, we demonstrate that the HA effect on midbrain development was selective for dopaminergic phenotype without affecting the generation of GABA neurons. In line with these findings, serotonin neurons, which are formed in the hindbrain very close to the midbrain-hindbrain boundary, were unaffected by HA.

In order to identify the cellular mechanism by which HA was exerting its action on decreasing TH + dopaminergic neurons, we co-injected HA with H_1_R or H_2_R antagonists. We found that only chlorpheniramine, a selective H_1_R antagonist, completely prevented the impairing action of HA over DA neurons. Our data support the hypothesis that HA action is on precursor dopaminergic cells by activation of H_1_R. We suggest that HA might be acting as a detrimental signal during early stages of DA specification. As we showed *in vitro*, incubation with HA leads to the elevation of Ca^2+^ in Nestin-positive VM NSPC, which might suggest that the mechanistic basis of HA effect over NSPC population affecting dopaminergic differentiation/specification involves intracellular Ca^2+^. Interestingly, Ca^2+^ signaling appears to control NSPC proliferation, regulating the switch from proliferation to neuronal differentiation [46]. For instance, in human NSPC, mobilization of IP_3_-dependent Ca^2+^ stores lengthens the cell cycle and increases the number of intermediate neural progenitors [34]. In this model, Ca^2+^ controls the duration of cell cycle by increasing the level of p53 protein, a known regulator of the cyclin-dependent kinase inhibitor p21 [47]. We have previously shown in cortical NSPC cultures the effect of HA on cell division patterns, evaluating asymmetric and symmetric divisions [28]. In the developing VM, proliferating cells are present in close to the ventricular zone [36]; we show that the number of mitotic cells is decreased by HA injection *in vivo*, and this could be a contributing mechanism for the decreased number of dopaminergic neurons.

The effects of artificially administering HA during VM development are clear, but we did not directly assess the participation of endogenous HA on dopaminergic development for example by decreasing its levels by inhibiting Histidine decarboxylase activity; nonetheless, the injection of H_1_ or H_2_ receptor antagonists alone did not modify TH staining, suggesting that the endogenous HA levels are not sufficient to affect dopaminergic development. Interestingly, the participation of HA in the pathogenesis of Parkinson’s disease in adult organisms has been suggested by previous work. For example, there is an increase in brain HA content in post-mortem putamen and *substantia nigra*[48] and HA blood levels are increased in parkinsonian patients [49]. Also, the number of histaminergic fibers innervating the *substantia nigra* is increased in individuals suffering Parkinson’s disease relative to controls [48],[50]. In addition to these suggestive data, there is experimental evidence showing that HA can affect differentiated dopaminergic neurons in the adult brain. Direct infusion of HA in the rodent *substantia nigra* leads to a selective damage of DA neurons [51]. One of the most widely used animal model for Parkinson’s disease is the intracerebral injection of the DA neurotoxin 6-hydroxydopamine in one hemisphere. When endogenous brain HA levels were increased or decreased, 6-hydroxydopamine-induced damage was potentiated or inhibited, respectively, assessed by TH immunostaining and by the rotational test after apomorphine administration. Supporting the participation of HA in the damage induced by this neurotoxin, the H_1_R antagonist pyrilamine, significantly, albeit transiently, decreased DA neuron degeneration; the H_2_R antagonist cimetidine, did not modify the 6-hydroxydopamine-induced damage [52].

Alternative mechanisms for the deleterious effects of HA on DA cells can include activation of microglial cells. In the adult *substantia nigra* injection of HA generates a microglial reaction, promoting dopaminergic cell death [51]. The possibility that microglia might contribute to DA degeneration in our model needs to be tested. There is evidence that microglia are present at this developmental stages [53], and that activated microglial cells reduce the number of neural precursor cells by phagocytosis in the developing cerebral cortex [54].

## Conclusion

Our findings reveal an unexpected role of HA receptor activation during embryogenesis in a specific neuronal phenotype. These effects are relevant for its significance as a possible therapeutic target in CNS pathologies that involve the interaction between both histamine and dopamine systems. In conclusion, our study contributes to the knowledge of factors participating on the generation and degeneration of DA neurons and reveals novel actions of HA during midbrain development, which might lead to design new and more specific protocols for the generation of dopaminergic neurons and future therapies for Parkinson’s disease.

## Methods

### Cell culture

Multipotent NSPC were isolated from E12-E12.5 embryos obtained from time-pregnant Wistar rats as described [27]. For simplicity, we only use E12 to designate these embryos and the same criterion applies for other embryonic stages. VM was dissected in Krebs solution then mechanically dissociated. Cells were recovered by centrifugation. Isolated cells were cultured on plastic ware treated with 15 μg/ml poly-L-ornithine (Sigma) and 1 μg/mL human Fibronectin (Invitrogen) in N2 medium (DMEM/F12 1:1, supplemented with 25 μg/L of human insulin, 30 nM sodium selenite, 100 μM putrescine, 20 nM progesterone and 100 mg/L of apotransferrin) containing 10 ng/mL of basic fibroblast growth factor (FGF-2; Peprotech). Cultures were maintained at 37°C in humidified 5% CO_2_/95% air atmosphere. These cultures were considered as passage (P) 0. When cultures reached 80% confluence, cells were detached with 0.1 mM EDTA in phosphate buffered saline (PBS) and re-plated to P1. Cells were maintained under proliferation conditions for 4 days in control (N2 medium + 10 ng/ml FGF-2) and experimental conditions that vary concentrations of HA (from 1 μM to 1 mM) with or without HA receptor antagonists. Differentiation was promoted by removal of FGF-2 during 6 additional days in control and experimental conditions. HA antagonists for H_1_R (1 μM chlorpheniramine, Sigma), H_2_R (30 μM cimetidine, Sigma) were added daily to HA-treated cells during proliferation and differentiation stages. For immunocytochemistry analysis, BrdU and TUNEL assays, cells were seeded at 1.5×10^4^/well in 24-well plates (Corning). For RNA extraction, cells were seeded at 3×10^5^/well in 6-well plates (Corning). HA dihydrochloride was used without neutralization.

### RNA extraction, RT-PCR and Real-Time RT-PCR

Total RNA was isolated from proliferating cultures, differentiated cultures, and midbrain tissue from ultrasound injected and control embryos using TRIZOL reagent (Invitrogen). One μg of RNA was reverse transcribed using dNTPs, 100 U of Superscript III Reverse Transcriptase, 50 μM of random hexamers and 40 U of Recombinant RNase Inhibitor (Invitrogen). PCR reactions were performed using 100 ng of cDNA, 2 U of recombinant Taq DNA polymerase (Invitrogen), 20 pmol of each oligonucleotide, 500 μM dNTPs (Sigma) and 1.5 mM MgCl_2._ PCR products were analyzed by electrophoresis to determine their molecular weights after ethidium bromide staining. The sequences of the PCR products were verified and correspond to the indicated HA receptor. Reactions with RNA in the absence of retrotranscription were included as negative controls. RNA extracted from adult rat cerebral cortex was used as a positive control for HA receptors. The forward (F) and reverse (R) primers listed in Table 1 were used.

**Table 1:** Primer sequences for detection of histaminergic receptors by end-point RT-PCR

**Gene**	**Sense primer**	**Antisense primer**
H_1_R	F: 5′-CTTCTACCTCCCCACTTTgCT-3′	R: 5′-TTCCCTTTCCCCCTCTTg-3′
H_2_R	F: 5′-TTCTTggACTCCTggTgCTgC-3′	R: 5′-CATgCCCCCTCTggTCCC-3′
H_3_R	F: 5′-CCAgAACCCCCACCAgATg-3′	R: 5′-CCAgCAgAgCCCAAAgATg-3′

These primers have been used previously by our group [27] and after retrotranscription and amplification, PCR products were extracted from the gel and sequenced, confirming that these sequences correspond to H_1_, H_2_ and the 3 isoforms of H_3_ receptors.

For qRT-PCR [55], we used 100 ng of the synthesized cDNA, 2.5 U of recombinant Taq DNA polymerase (Invitrogen), 0.4 mM of each oligonucleotide and dNTPs, 1.5 mM MgCl_2_ and 5 μM Syto 9 (Invitrogen). Quantitative analysis of cDNA amplification was performed using the Rotor Gene 6000 System (Corbett Life Science). Melting curves were performed in every reaction to ensure that only one product was present. GAPDH amplification was used as loading control for all reactions and every transcript value was normalized to GAPDH expression. The 2exp (−ΔΔCt) method was used to determine the relative expression of each gene compared to control condition. The primers listed in Table 2 were used.

**Table 2:** Primer sequences for detection of transcripts by qRT-PCR

**Gene**	**Sense primer**	**Antisense primer**
β-III Tubulin	F: 5′-ggCCTTTggACACCTATTCA-3′	R: 5′-TgCAggCAgTCACAATTCTC-3′
TH	F: 5′-CCACTggAggCTgTggTATT-3′	R: 5′-CCgggTCTCTAAgTggTgAA-3′
GAPDH	F: 5′-gTggACCTCATggCCTACAT-3′	R: 5′-ggATggAATTgTgAgggAgA-3′

### Western blot

Cells were cultured and seeded at 3.5×10^4^/well in 6-well plates (Corning) in control and experimental conditions, following standard protocols [27],[56]. Briefly, cells were homogenized in lysis buffer (25 mM Tris–HCl pH 7, 1% IGEPAL, 100 mM NaCl) supplemented with protease inhibitors (Complete mini, Roche). Proteins were obtained by centrifugation at 13 800 g at 4°C for 15 min, and quantified by Bradford assay (BioRad). Proteins (30 μg) were resolved on 8% sodium dodecyl sulfate–polyacrylamide gel electrophoresis and transferred to nitrocellulose membranes (Amersham Bioscience). The membranes were blocked with 5% non-fat milk and incubated overnight with primary antibodies. Pre-stained markers (Invitrogen) were included for size determination. The following antibodies were used: rabbit anti-rat H_1_R polyclonal antibody (1:1500, Santa Cruz Biotechnology); goat anti-rat H_2_R polyclonal antibody (1:1500, Santa Cruz Biotechnology); rabbit anti-Lmx1a polyclonal antibody (1:100, Abcam); rabbit anti-Lmx1b polyclonal antibody (1:100, Abcam); rabbit polyclonal anti-tyrosine hydroxylase (TH, 1:1000; Pel-freez) and rabbit anti-Pitx3 polyclonal antibody (1:500, Zymed). Membranes were washed and incubated with corresponding horseradish peroxidase-coupled secondary antibodies (1:10 000, Santa Cruz Biotechnology) for 2 hours. Immunoreactive bands were detected using enhanced chemiluminescence method (Amersham Bioscience) and developed on photographic film (Kodak).

### Immunocytochemistry

Cells were fixed with 4% paraformaldehyde in PBS, pH 7.4 at the end of proliferation and differentiation stages. Immunocytochemistry assays were performed following standard protocols [57]. Briefly, cells were permeabilized and blocked for 1 hour with 0.3% Triton X-100 and 10% normal goat serum (NGS) in PBS. Cells were incubated overnight at 4°C with the following primary antibodies diluted in PBS containing 10% NGS: rabbit polyclonal anti-β-III Tubulin (1:2000, Covance); rabbit polyclonal anti-glial fibrillary acidic protein (GFAP; 1:2000, DAKO); mouse monoclonal antibody anti-microtubule associated protein 2 (MAP2; 1:500, Chemicon); mouse monoclonal anti-Nestin (1:100, Developmental Studies Hybridoma Bank); rabbit anti-Nestin (1:1000, Covance); rabbit polyclonal anti-TH (1:1000; Pel-freez); rabbit polyclonal anti-Sox2 (1:200; Millipore). Appropriate Alexa-Fluor secondary antibodies were used diluted in PBS (1:1000; Molecular Probes) containing 10% NGS. Nuclei were stained with Hoechst 33258 (1 ng/mL; Sigma). Immunostainings were analyzed with an epifluorescence microscope (Nikon, Eclipse TE2000-U) and photographed with a Nikon digital camera (DMX1200 F). Negative controls were performed in the absence of primary antibodies and showed no unspecific staining.

### BrdU incorporation assay

Cells treated with FGF-2 were incubated for 3 hours with 10 μM 5-bromo-2-deoxyuridine (BrdU, Roche), washed with medium and fixed with 4% paraformaldehyde in PBS 21 h later [27]. After fixation, cells were incubated with 1 N HCl for 30 min at 25°C, and neutralized by washing with 0.1 M borate buffer, pH 8.5. Preparations were blocked for 1 h with 10% NGS (Invitrogen) and 0.3% Triton X-100 (Sigma) in PBS. Cells were incubated overnight at 4°C with monoclonal rat anti-BrdU antibody (1:100; Accurate) followed by three washes with 1% bovine serum albumin in PBS. Alexa-Fluor 488 goat anti-rat IgG was added (1:1000, Invitrogen) in blocking solution for 1 h at RT and washed three times with PBS. Nuclei were stained with Hoechst 33258 (1 ng/mL; Sigma).

### TUNEL assay

Proliferating and differentiated cells were fixed with 4% paraformaldehyde in PBS, and subsequently washed three times with PBS, pH = 7.4 [27]. For detection of apoptotic cells, the Terminal deoxytransferase-mediated deoxyuridine triphosphate nick-end labeling kit (TUNEL, InSitu Cell Death Detection Kit, Roche Diagnostics) was used to evaluate the effect of HA on cell death. Apoptotic cells were visualized by fluorescence microscopy.

### Fluoro Jade staining

Fluoro Jade was used to label and quantify degenerating neurons in the VM. The sections were mounted on slides and air-dried. The slides were dipped in absolute ethanol for 3 min followed by 1 min in 70% ethanol and washed in deionized water for 1 min. Tissue was then oxidized with a 0.06% KMnO_4_ solution diluted in PBS for 15 min, followed by three rinses with deionized water. From this point onwards, the slides were protected from light. Tissue sections were stained with a 0.001% solution of Fluoro-Jade in 0.1% acetic acid for 30 min. Slides were rinsed with water and air-dried. The slides were cleared in xylene and coverslipped with DPX mounting medium [58],[59].

### Cell counting

Cell counts from BrdU, immunocytochemistry, TUNEL, phosphorylated histone H3 (pHH3) and Fluoro Jade experiments were performed from digital pictures. Quantification of cells was performed by using the ImageJ software considering the total number of Hoechst-stained nuclei (total cells), in at least 10 random fields from a minimum of 3 independent experiments.

### Intracellular Ca^2+^ imaging

Proliferating VM NSPC in suspension were loaded with the Ca^2+^-sensitive fluorescence indicator Fura-2 AM (Invitrogen) at a final concentration of 5 μM at 37°C for 45 min [29]. Loaded cells were centrifuged and seeded at 1×10^5^ cells on 3.5 cm glass bottom dishes (MatTek Corp) treated with 15 mg/mL poly-L-ornithine (Sigma) and 1 μg/ml human Fibronectin (Invitrogen) in N2 medium. After 2 hours, attached cells were incubated in Hank’s solution (137 mM NaCl, 5 mM KCl, 0.3 mM NaH_2_PO_4_, 0.8 mM MgSO_4_, 1 mM MgCl_2_, 5 mM glucose, 2 mM glutamine, 10 mM Tris–HCl without CaCl_2_; pH 7.4) at 37°C for 10 minutes. Subsequently, Hank’s solution was removed and new solution with 5 mM CaCl_2_ was added. UV excitation was set to alternate between 340 and 380 nm and the emission was monitored at 510 nm in a Carl Zeiss microscope. Images and the emission ratio generated between both excitations wavelengths (*R* = *E*340/*E*380) were obtained and analyzed using AxioVision software.

### Ultrasound guided injections

We used timed-pregnant Wistar rats with embryos at gestational ages E10, E12, E14 and E16 to perform ultrasound-guided injections. All animal procedures were approved by the Instituto de Fisiología Celular Animal Use and Care Committee and conformed to Mexican and NIH guidelines. All injections were performed by duplicate or triplicate in different embryos from the same dam, and repeated at least in 3 different pregnant rats. Control and experimental animals were included in the same pregnant rat, but to make sure that the different conditions do not mix, each group was evaluated in a single uterine horn. The results were reproducible when we analyzed embryos from different dams. To perform ultrasound guided injections [40], we used an MHF-1 Ultraview UltraSound System (E-Technologies) with a focal distance of 7 mm for imaging. Rats were deeply anesthetized in a chamber and then maintained with a facemask to administer inhaled sevoflurane (5% for induction and 1% for maintenance). We performed midline laparotomy, uterine horns were carefully exposed and the number of embryos was recorded. Intraventricular injections were performed through the uterine wall using pulled microcapillaries (borosilicate glass, Sutter Instruments). The glass needles were visualized in the ultrasound imaging system and the verification of the site of injection in the lateral ventricles was made during the procedure by observing the liquid going out of the needle. HA, chlorpheniramine (H_1_R antagonist) or cimetidine (H_2_R antagonist) were injected on the ventricular lumen at different doses in a final volume of 2 μl aided by an automatic injector (Quintessential injector, Stoelting). The injected amount was of HA dihydrochloride, which was used without neutralization. DMSO and Cell Tracker (Invitrogen) injections were performed as controls. Once the desired number of embryos was injected, the uterus was repositioned in its physiological site. The incisions of the abdominal muscle and skin were stitched with separate sutures. Finally we applied Buprenorphine as analgesic (0.1 mg/kg, Pisa Laboratories) and animals were monitored and left to recover. Two days after the injections, pregnant rats were euthanized by overdose of the anesthetic Sodium pentobarbital (Pfizer), and identified embryos were recovered for analysis. We performed the following number of successfully injected E12 embryos: vehicle: 48; HA: 12; chlorpheniramine: 9; cimetidine: 9; HA plus chlorpheniramine: 9; HA plus cimetidine: 9. The number of dams used was 18.

### Immunohistochemistry

Embryos were removed and transferred to PBS. Subsequently, the embryos were fixed by immersion overnight in 4% PFA in PBS at 4°C. Thereafter, the tissues were cryoprotected overnight in 30% (v/v) sucrose, embedded in Tissue Freezing Medium (Tissue-Tek, Zakura Finetek) and frozen. Sagittal and coronal sections (20 μm) were obtained on a cryostat (Leica). For immunohistochemistry, slides were rehydrated in PBS. Tissue sections were permeabilized and blocked for 1 hour at RT with 0.3% Triton X-100 and 10% NGS in PBS. Slides were incubated overnight at 4°C with the following primary antibodies diluted in PBS containing 10% NGS: rabbit polyclonal anti-pHH3 (1:250, Cell Signaling), rabbit polyclonal anti-β-III Tubulin (1:1000, Babco Covance); mouse monoclonal anti-Nestin (1:100, Developmental Studies Hybridoma Bank); rabbit polyclonal anti-tyrosine hydroxylase (TH; 1:1000, Pel-freez); rabbit polyclonal anti-GAD65/67 (1:500, Millipore); rabbit polyclonal anti-serotonin (1:500, Sigma). Subsequently, the sections were washed three times for 5 minutes in PBS and incubated with the secondary antibodies Alexa-Fluor 488 anti-rabbit IgG and Alexa-Fluor 568 anti-mouse IgG (1:500; Molecular Probes) in PBS containing 10% NGS for 2 hours. Nuclei were stained with Hoechst 33258 (1 ng/mL). After three further washes in PBS, the sections were mounted in Aqua Poly/Mount (Polysciences, Inc.) Immunostainings were analyzed with an epifluorescence microscope (Nikon, Eclipse TE2000-U) and photographed with a Nikon digital camera (DMX1200 F). Some images were acquired with a FV1000 Olympus confocal microscope. Negative controls were performed in the absence of primary antibodies and showed no unspecific staining.

### Statistical analysis

We performed 3–5 independent experiments, defined as duplicate or triplicate experiments from a different pregnant rat. All data are presented as mean ± standard error of mean (S.E.M). One-way ANOVA was performed for statistical analysis and multiple comparisons between treated and control groups were made using the post-hoc Student-Newman-Keuls test. Differences were considered statistically significant at P ≤ 0.05. Graphs were generated using GraphPad Prism software.

## Competing interests

The authors declare no competing financial interests.

## Authors’ contributions

IEA wrote the manuscript, analyzed the data and performed most of the experiments with help from FVR. AMH contributed to the design of experiments and discussion of results. RLG helped to perform intrauterine injections, analysis and discussion of results. DC and JADC contributed to set up techniques reviewed the manuscript and took part in the discussion of results. All authors read and approved the final manuscript. IV contributed to experiment design/supervision, reviewed the manuscript and obtained funding.
